# Supplementary material for: Lack of p62 Impairs Glycogen Aggregation and Exacerbates Pathology in a Mouse Model of Myoclonic Epilepsy of Lafora
Source: Mol Neurobiol. 2021 Dec 28;59(2):1214–29. doi: 10.1007/s12035-021-02682-6 (PMC8857170; doi:10.1007/s12035-021-02682-6)
Supplement: Supplementary file 1 — Supplementary file1 (PDF 3492 KB) Supplementary Figure 1. p62 deletion prevents glycogen aggregation in heart. A. Histological localization of LBs in heart. Periodic acid-Schiff staining (PAS) is shown for the indicated tissues of 11-month-old malinKO and 11-month-old malinKO+p62KO littermates. Scale bar=25 µm. B. Representative immunostaining of p62 and MGS in heart. Scale bar=25 µm Supplementary Figure 2. Additional morphology parameters of neuronal LBs and astrocytic CAL. Quantifications of the indicated morphological parameters in neuronal LBs and astrocytic CAL. A linear mixed model analysis was performed for statistical analysis. N=6 animals per genotype. Adjusted p-values. Area: astrocytic p=0.657; neuronal p=0.269. Perimeter: astrocytic p=0.952; neuronal p=0.592. MGS mean intensity: astrocytic p=0.771; neuronal p=0.388. Centroid Xm: astrocytic p=0.392; neuronal p=0.959. Centroid Ym: astrocytic p=0.646; neuronal p=0.809. Min caliper: astrocytic p=1; neuronal p=0.162. Supplementary File 3. Raw and adjusted p-values of the pairwise comparisons in Figure 7A. Supplementary File 4. SYBR green primers used in Figure 7. [file 12035_2021_2682_MOESM1_ESM.pdf]

Supplementary Figure 1

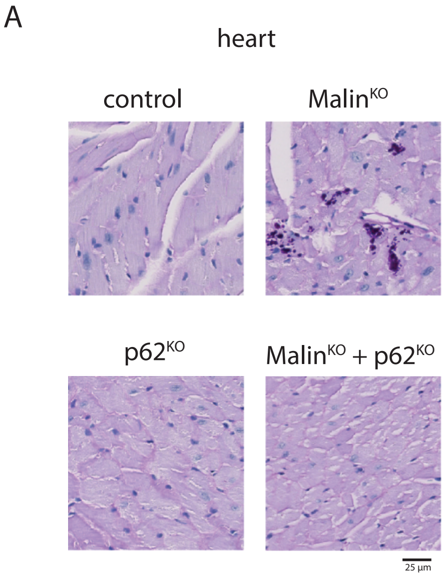

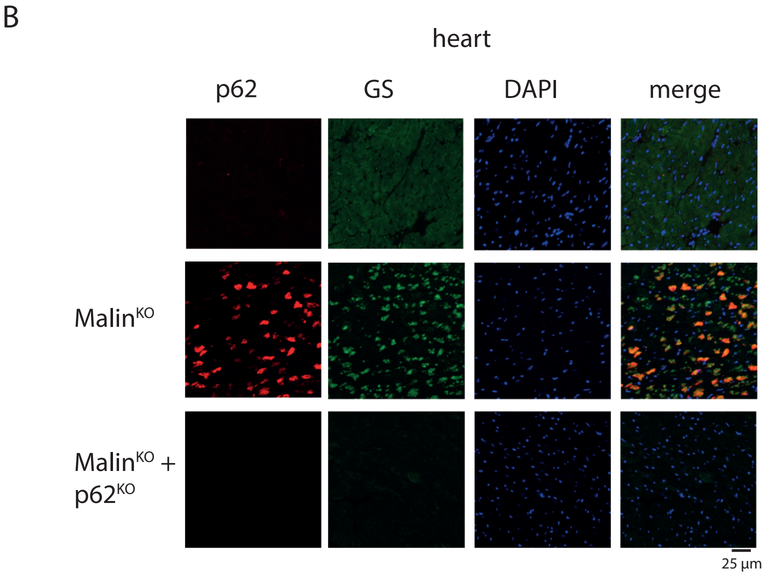

Supplementary Figure 2

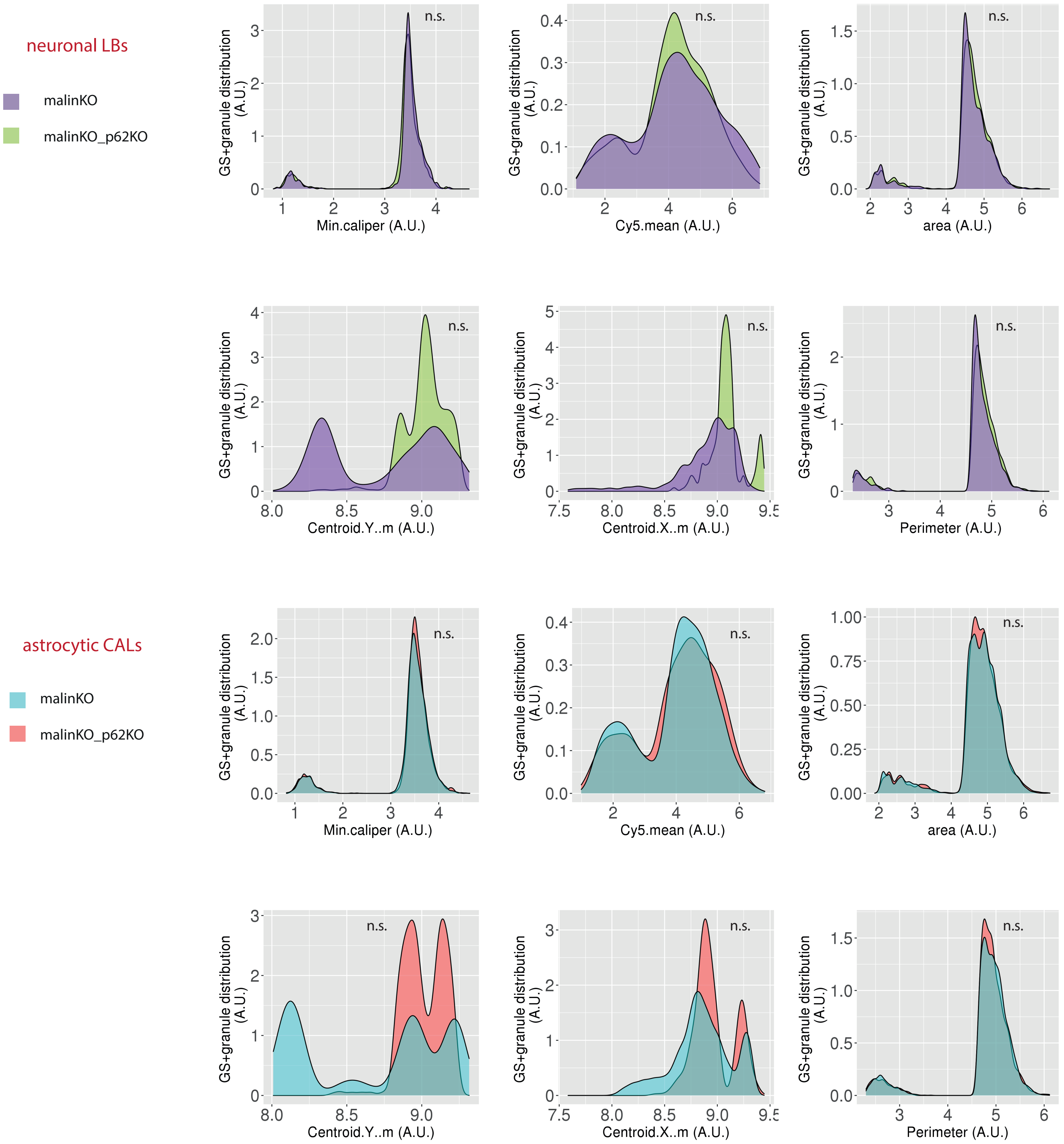

Supplementary File 3

raw p-values

gene	p62KO -CTRL	malinKO -CTRL	malinKO+p62KO -CTRL	malinKO+p62KO -malinKO
iNOS	0,000627985	3,28E-05	3,72E-05	0,81796078
IL6	3,83E-06	7,50E-09	7,02E-13	0,29406075
IFN-g	0,024965437	8,09E-05	1,49E-06	0,576956352
C1q	0,002368484	1,66E-07	8,23E-09	0,804516837
TREM2	0,013795538	9,47E-10	1,56E-10	0,975476864
IL12	0,180660635	0,700943651	0,025707614	0,012892388
IL18	0,649799372	0,925090468	0,019875824	0,020941161
IL23	0,096005457	0,150064159	0,081135417	0,826105466
CD206	0,183028442	2,12E-05	0,005927228	0,101948059
IL4	0,078967515	0,00756228	0,930594325	0,005889722
IL10	0,172617973	0,051169739	0,304207368	0,457743055
TGFb	0,045404599	1,98E-05	3,61E-07	0,566873938
C3	0,257200748	4,25E-13	0	0,096674678
CCL2	0,04324087	8,49E-11	0	0,061833936
CXCL10	0,855087833	1,21E-10	8,90E-10	0,75650904
CXCL12	0,182336696	0,000693553	1,25E-11	0,000732561
LCN2	0,419700063	2,49E-08	3,82E-10	0,492117664
Arg1	0,868773282	0,00041276	0,751981875	0,001300992
Cd109	0,050671929	0,123805406	0,008760502	0,279134297
Cd14	0,076345881	0,020858112	7,18E-06	0,029430864
Cp	0,987136866	0,002233728	1,56E-05	0,18413109
S100a10	0,785335781	0,13050126	0,277190034	0,670490581
TNFa	0,719892712	0,002766171	6,65E-05	0,319335641
IL13	0,687529219	0,034313818	0,957773761	0,038828346
IL1b	3,05E+05	7,94E+00	6,76E-01	6,16E+05

adjusted p-values

gene	p62KO -CTRL	malinKO -CTRL	malinKO+p62KO -CTRL	malinKO+p62KO -malinKO
iNOS	0,00224542	0,00012611	0,00017007	0,99367684
IL6	1,10E-05	2,38E-08	1,48E-12	0,65056
IFN-g	0,0833311	0,0003818	3,79E-06	0,922294
C1q	0,0089913	4,52E-07	2,41E-08	0,9923234
TREM2	0,047681	2,01E-09	4,17E-10	0,999984
IL12	0,460676	0,972368	0,085889	0,0447
IL18	0,955528	0,999558	0,067333	0,070732
IL23	0,27596	0,39789	0,23919	0,99449
CD206	0,469956	7,20E-05	0,021667	0,294061
IL4	0,23363	0,027158	0,999648	0,021412

IL10	0,44436	0,15988	0,66558	0,83882
TGFb	0,14353	7,65E-05	2,76E-06	0,91657
C3	0,59361	1,10E-12	< 2.22e-16	0,27707
CCL2	0,13716	1,95E-10	< 2.22e-16	0,18856
CXCL10	0,99678	2,68E-10	2,05E-09	0,98483
CXCL12	0,4620595	0,002663	2,76E-11	0,0027137
LCN2	0,80204	9,52E-08	5,40E-09	0,86601
Arg1	0,997609	0,001512	0,983967	0,004792
Cd109	0,158076	0,340153	0,031052	0,627567
Cd14	0,22646	0,070539	2,22E-05	0,096618
Cp	0,9999977	0,0082964	5,31E-05	0,4609317
S100a10	0,98958	0,3551	0,62464	0,96266
TNFa	0,97696141	0,00996043	0,00024165	0,68483843
IL13	0,9683	0,11141	0,99992	0,12496
IL1b	0.66467	3,96E-01	2,64E-02	0.94127

Supplementary File 4

TARGET	FORWARD	REVERSE
TREM2	GACCTCTCCACCAGTTTCTCC	TACATGACACCCTCAAGGACTG
TGFb	TGACGTCACTGGAGTTGTACGG	GGTTCATGTCATGGATGGTGC
S100a10	CTGCTCACAAGAAGCAGTGG	CCTCTGGCTGTGGACAAAAT
IL-4	GGTCTCAACCCCCAGCTAG	GCCGATGATCTCTCTCAAGTGAT
IL13	GGGTGACTGCAGTCCTGGCT	GTTGCTCAGCTCCTCAATAAGC
IL10	CCAAGCCTTATCGGAAATGA	TTTTCACAGGGGAGAAATCG
CD206	CAAGGAAGGTTGGCATTTGT	CCTTTCAGTCCTTTGCAAGC
Cd109	GCAGCGATTTCGATGTCCAC	CACAGTCGGGAGCCCTAAAG
Arg1	CCTCGAGGCTGTCCTTTTGA	TTTTAGGGTTACGGCCGGTG
Tnfa	CTTGATGGTGGTGCATGAGA	TGTGCTCAGAGCTTTCAACAA
Lcn2	CACACTCACCACCCATTCAG	CCAGTTCGCCATGGTATTTT
iNOS	GGCAGCCTGTGAGACCTTTG	CATTGGAAGTGAAGCGTTTCG
IL6	TCCTACCCCAACTTCCAATGCTC	TTGGATGGTCTTGGTCCTTAGCC
IL23	TGTGCCCCGTATCCAGTGT	CGGATCCTTTGCAAGCAGAA
IL1β	ACGGACCCCAAAAGATGAAG	TTCTCCACAGCCACAATGAG
IL12	GGAAGCACGGCAGCAGAATA	AACTTGAGGGAGAAGTAGGAATGG
IFN-γ	TCAAGTGGCATAGATGTGGAAGAA	TGGCTCTGCAGGATTTTCATG
CXCL10	GACCATCAAGAATTTAATGAAAGCG	CCATCCACTGGGTAAAGGG
Cp	AGTGTATAGAGGATGTTCCAGGTCA	TGTGATGGGAATGGGCAATGA
Cd14	GCTTCAGCCCAGTGAAAGAC	GGACTGATCTCAGCCCTCTG
CCL2	GAGTAGGCTGGAGAGCTACAAGAG	AGGTAGTGGATGCATTAGCTTCAG
C1q	CCCTGGTAAATGTGACCCTTTT	TCTGCACTGTACCCGGCTA
C3	TCCTGAACTGGTCAACATGG	AAACTGGGCAGCACGTATTC
Ccl12	ACCATCAGTCCTCAGGTATTGG	TTCCGGACGTGAATCTTCTG
IL18	GCCTCAAACCTTCCAAATCA	TGGATCCATTTCCTCAAAGG
